# Age-Dependent Evolution of the Yeast Protein Interaction Network Suggests a Limited Role of Gene Duplication and Divergence

**DOI:** 10.1371/journal.pcbi.1000232

**Published:** 2008-11-28

**Authors:** Wan Kyu Kim, Edward M. Marcotte

**Affiliations:** Center for Systems and Synthetic Biology, Institute for Cellular and Molecular Biology, University of Texas at Austin, Austin, Texas, United States of America; National Cancer Institute United States of America and Tel Aviv University, Israel

## Abstract

Proteins interact in complex protein–protein interaction (PPI) networks whose topological properties—such as scale-free topology, hierarchical modularity, and dissortativity—have suggested models of network evolution. Currently preferred models invoke preferential attachment or gene duplication and divergence to produce networks whose topology matches that observed for real PPIs, thus supporting these as likely models for network evolution. Here, we show that the interaction density and homodimeric frequency are highly protein age–dependent in real PPI networks in a manner which does not agree with these canonical models. In light of these results, we propose an alternative stochastic model, which adds each protein sequentially to a growing network in a manner analogous to protein crystal growth (CG) in solution. The key ideas are (1) interaction probability increases with availability of unoccupied interaction surface, thus following an anti-preferential attachment rule, (2) as a network grows, highly connected sub-networks emerge into protein modules or complexes, and (3) once a new protein is committed to a module, further connections tend to be localized within that module. The CG model produces PPI networks consistent in both topology and age distributions with real PPI networks and is well supported by the spatial arrangement of protein complexes of known 3-D structure, suggesting a plausible physical mechanism for network evolution.

## Author Summary

Proteins function together forming stable protein complexes or transient interactions in various cellular processes, such as gene regulation and signaling. Here, we address the basic question of how these networks of interacting proteins evolve. This is an important problem, as the structures of such networks underlie important features of biological systems, such as functional modularity, error-tolerance, and stability. It is not yet known how these network architectures originate or what driving forces underlie the observed network structure. Several models have been proposed over the past decade—in particular, a “rich get richer” model (preferential attachment) and a model based upon gene duplication and divergence—often based only on network topologies. Here, we show that real yeast protein interaction networks show a unique age distribution among interacting proteins, which rules out these canonical models. In light of these results, we developed a simple, alternative model based on well-established physical principles, analogous to the process of growing protein crystals in solution. The model better explains many features of real PPI networks, including the network topologies, their characteristic age distributions, and the spatial distribution of subunits of differing ages within protein complexes, suggesting a plausible physical mechanism of network evolution.

## Introduction

Life is highly organized at all levels of molecules, cells, tissues, and organisms, and such relationships among biological entities are often represented as networks, with vertices representing e.g. genes or proteins, and edges representing e.g. physical protein interactions, transcriptional regulation, or metabolic reactions. The topology of biological networks shows many interesting characteristics, such as scale-free topology (power-law or broad degree distribution) and hierarchical modularity (reviewed in [1]). These properties are believed to be the basis of functional modularity, error-tolerance, and stability [2]–[5] characteristic of many biological networks.

One important question is thus how these important network architectures originate, and what driving forces underlie the observed networks. It has not been clear whether network architecture results from the mosaic sum of each gene or protein's inherent properties, such as *stickiness* or *interactive promiscuity* [6],[7], or from a stochastic mechanism underlying network evolution, in which the trajectory of network evolution is conditioned on the previous state of the network [8]. This problem has been of wide interest because it raises fundamental questions about design principles of molecular networks and the role of natural selection in the evolution of network structure [9].

Initially, Barabási and Albert proposed a preferential attachment rule as a general mechanism to generate scale-free networks [8]. In this model, a newly introduced node is more likely to be attached to highly connected nodes, resulting in a power-law degree distribution. In a network of protein-protein interactions (PPI), gene duplication and divergence (DD) is most popularly thought of as the origin of the scale-free topology of protein interaction networks [10]–[15]. In the DD model, the degree of a node increases mainly by having duplicate genes as its neighbors. Therefore, the preferential attachment rule is achieved implicitly, with highly connected nodes having more chance to have duplicate genes as their neighbors [1]. The DD model is also shown to generate hierarchically modular networks under certain conditions [16].

Although the DD model generates scale-free and modular networks, it has drawbacks that must be noted if it is to be considered a main mechanism for PPI network evolution. Primarily, only a small fraction of duplicate genes effectively contribute to the overall network topology. The key feature of the DD model originates from the fact that duplicate genes share a certain number of interaction partners. However, the interaction patterns of duplicate genes diverge rapidly [17], and the vast majority of gene duplicates are shown to share no interaction partners [18]–[20]. Some duplicates, in fact, may have diverged so extensively that they can no longer be detected by sequence homology. These distant duplicates would share even fewer interaction partners, and thus they are essentially indistinguishable from non-duplicate pairs in terms of interaction patterns.

To better understand the evolution of PPI networks, we analyzed a non-topological property—the age of each protein as estimated based upon the taxonomic distribution of its constituent domains [21],[22]—and observe that yeast PPI networks show a unique interaction density pattern between different protein age groups. The density pattern of the yeast PPI network was compared with those generated by canonical network evolution models—preferential attachment (the Barabási-Albert model), duplication-divergence (DD), and anti-preferential attachment (AP). Each model generates a unique interaction density pattern between the age groups; thus, the validity of the models could be effectively discriminated. Using this test, we observe that none of the canonical models are consistent with real yeast PPI networks. The age-dependent interaction density pattern nonetheless suggests growth by a stochastic process. We therefore propose an alternative model called the crystal growth (CG) model, which is based upon known physical and chemical principles and shows good agreement with real PPI networks in both topological and age properties as well as the 3-D subunit configurations of protein complexes.

## Results

### Interaction Density Patterns between Protein Age Groups

First, we introduce the basic attachment rules of protein-protein interactions. The interaction densities, D_m,n_, between two protein age groups (m,n) show unique patterns depending upon the attachment rule. Three basic rules are considered—random attachment (RA), preferential attachment (PA) by Barabási and Albert [8],[23], and anti-preferential attachment (AP). Here, we consider three protein age groups (G1, G2, and G3, from oldest to youngest), and assume a fixed number of new connections (ΔE) are made between a newly introduced node and the existing nodes as a network grows.

**Figure 1:**
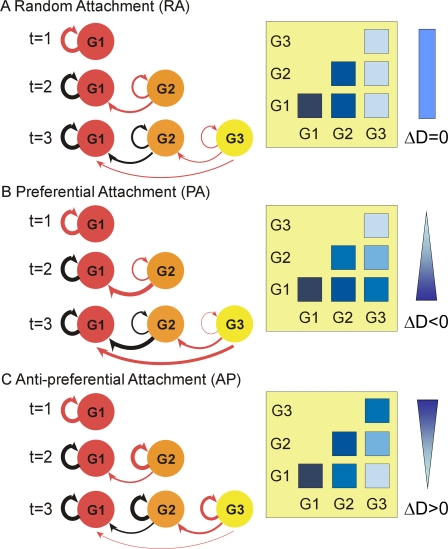
Interaction density (D) patterns depend upon the attachment rule. The protein age groups G1, G2, and G3 emerge at times t = 1, 2, and 3, respectively. In all cases, the first age group, G1, makes intra-group connections at t = 1. (A) In the random attachment (RA) model, G2 makes connections to G1 and within G2 with an equal probability at t = 2, showing that D_1,1_ = D_1,2_. Similarly, G3 makes connections to G1, G2, and within G3 (D_1,3_ = D_2,3_ = D_3,3_). The interaction densities between protein age groups are shown in the right panel. (B) In the preferential attachment (PA) model, G2 attaches more frequently to G1 than within G2 because, on average, G1 is more connected (D_1,2_>D_2,2_). At t = 3, G3 is preferentially connected to older groups in the order of G1>G2>G3 (D_1,3_>D_2,3_>D_3,3_). (C) In anti-preferential attachment (AP), the interaction density shows the reverse pattern to PA. Because a new node prefers less-connected nodes or younger groups, the density pattern shows D_1,2_<D_2,2_ and D_1,3_<D_2,3_<D_3,3_. Therefore, the interaction density (D) decreases in AP but increases in PA from top to bottom in the right panel.

In the RA model, a new node is randomly connected to existing nodes with equal probabilities. Initially, at time t = 1, the first age group, G1, makes only intra-group connections. Then a new group, G2, is introduced and connected randomly either to G1 (inter-group) or within G2 (intra-group). In the RA model, the expected interaction density, D, is the same between D_1,2_ and D_2,2_. Similarly, G3 connects to G1, G2, and within G3, showing the pattern of D_1,3_ = D_2,3_ = D_3,3_. More generally, the RA model shows a pattern of D_m,n_ = D_m+1,n_ (m<n) (Figure 1A). In the PA mode, new proteins are preferentially connected to highly connected nodes. Thus, G2 proteins are more likely to be linked to G1 than G2 because G1 proteins have previously made connections and have a higher average degree. Likewise, G3 proteins are more likely to be connected to older groups, showing D_1,3_>D_2,3_>D_3,3_. Thus the typical pattern of the PA model is D_m,n_>D_m+1,n_ (m<n) (Figure 1B). The AP model shows an inverse pattern to the PA model, D_m,n_<D_m+1,n_ (m<n), because new nodes prefer to connect to less-connected nodes (Figure 1C).

As a measure of age-dependency of interaction density, ΔD is defined as the average value of D_m+1,n_ - D_m,n_ (m<n) (see Methods). A positive ΔD indicates that protein interactions are more likely between similar age groups. The sign of ΔD effectively discriminates each model—it is positive in PA, negative in AP, and near zero in the RA model.

### Characterization of the Yeast PPI Network

We collected two independent sets of yeast PPIs - literature curated (LC) and high-throughput (HTP) PPIs, using the method of Batada *et al.* [23],[24] (Dataset S1 and Dataset S2) and inspected both the network topology and the age-dependency of interaction density. The number of nodes, N (proteins) and edges, E (interactions) in the LC and HTP networks are N_LC_ = 3268, E_LC_ = 12058 and N_HTP_ = 2488, E_HTP_ = 6766 respectively. The union (LC+HTP) of the two networks has 3780 nodes and 16505 edges. As HTP and LC+HTP show highly similar characteristics (Figure S2) as well as the original set by Batada *et al*. [23],[24], we mainly discuss the LC data set as the yeast PPI network (PPI_yeast_) here. The recently compiled set (Y2H-union) by Vidal and colleagues [25] from large-scale yeast two-hybrid experiments showed the same trend (Figure S2).

**Figure 2:**
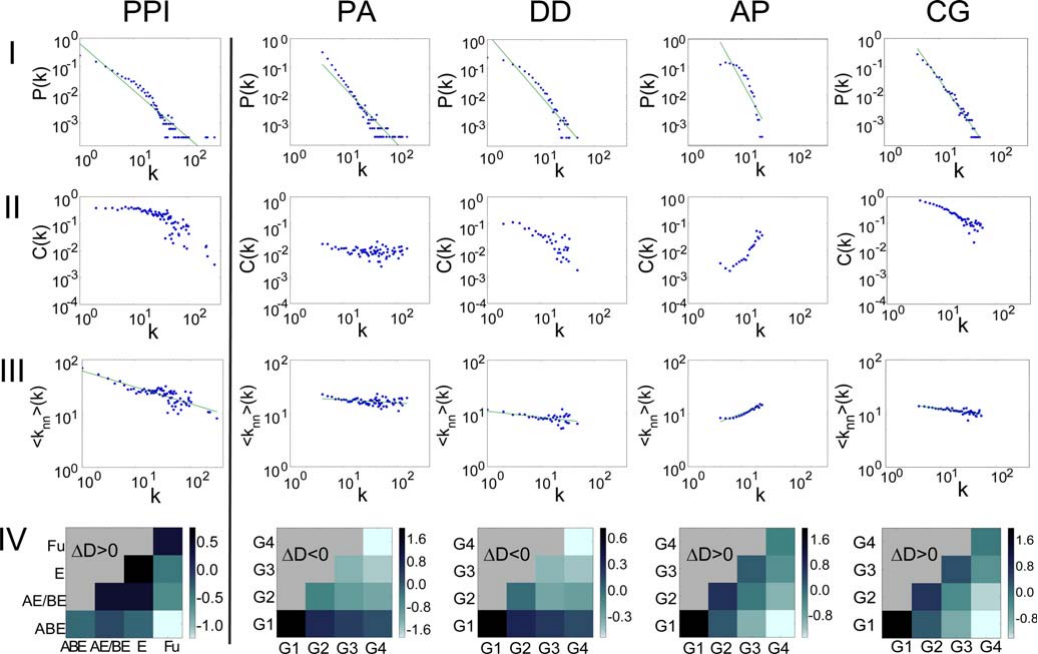
The network properties of the yeast PPI network are compared with the different models for network evolution. None of the canonical models (PA, DD, and AP) were compatible with the real PPI_yeast_ in terms of both topology and the age-dependency of interaction density. Only the CG model shows similar characteristics to the PPI_yeast_ for all the network properties tested. The plots in each row, I-IV, indicate (I) the degree distribution P(k), (II) the clustering coefficient C(k), (III) the average degree of nearest neighbors <k_nn_>(k), and (IV) the interaction density pattern (ΔD) between protein age groups. In the yeast PPI, the network shows a scale-free degree distribution, hierarchical modularity, and dissortative mixing properties (negative correlation in rows I-III, respectively). In row IV, the interaction density tends to be dense within the same group (diagonal) and sparse between different age groups (off-diagonal) in each column with positive ΔD, similar in pattern to the anti-preferential attachment (AP) in Figure 1C. In the PA model, the resulting network is scale-free (I) and slightly dissortative (III), similar to the PPI_yeast_. However, it is not hierarchically modular (II) and shows an inverse pattern of negative ΔD. In the DD model, the resulting network is scale-free (I), dissortative (III), and also hierarchically modular but not as highly as the PPI_yeast_ (II). It shows an inverse pattern of negative ΔD as the PA model. In the AP model, the resulting network is highly different from the PPI_yeast_, showing non scale-free, non hierarchically modular, and non dissortative structure (I-III), although the interaction density pattern (ΔD>0) is similar (IV). In the CG model, the network shows highly similar network characteristics to PPI_yeast_ in both topology (I-III) and interaction density (IV). The number of nodes is N = 3,000 in all cases. The average degree is <k> = 8 in the PA, AP, and CG models, and in the DD model the parameters are set as p = 0.1 and q = 0.6, where the resulting average degree is <k>≈4.

The PPI_yeast_ recapitulates known topological features such as a scale-free degree distribution, hierarchical modularity, and degree-dissortative mixing property [8], [26]–[28], which were characterized by the various network property indices shown in the first column (PPI) in Figure 2 (summarized in Table S1). The probability of a node having degree k shows a scale-free or power-law degree distribution in P(k) ∼ k^−γ^ plot (the row I in Figure 2). The PPI_yeast_ is shown to be highly modular, with a high degree of clustering coefficient, C and modularity index, Q defined by Newman [29]. In particular, the PPI_yeast_ has a scaling property in C(k) ∼ k^−β^ plot (β>0), suggesting hierarchical modularity [27] (the row II in Figure 2). In a dissortative network, high-degree nodes (hubs) tend to connect with low-degree nodes and hub-hub interactions are suppressed, as called the Maslov-Sneppen rule [30]. The degree-dissortativity was characterized by a negative correlation in <k_nn_>(k) ∼ k^δ^ (δ<0) plot (the row III in Figure 2), where <k_nn_>(k) is the average degree of the nearest neighbors of the nodes with degree k.

Surprisingly, the interaction density of PPI_yeast_ is also highly age-dependent. Yeast proteins were assigned to one of the age groups ABE, AE/BE, E and F depending on the taxonomic distribution of constituent domains among archaea (A), bacteria (B), eukaryote (E) and fungi (F) (see Methods, Figure S1). We measured the interaction density between the age groups and observe a positive ΔD similar to AP model (the row IV in Figure 2). The pattern of positive ΔD is highly robust regardless of the sources of data (LC, HTP and LC+HTP) and the random addition or deletion of edges, e.g. by 50%. It suggests that the positive ΔD is a genuine feature of PPI_yeast_.

### Simulation of Canonical Network Growth Models

We next simulated PPI network evolution using the three canonical models—PA (preferential attachment), DD (duplication and divergence), and AP (anti-preferential attachment) and tested compatibility with PPI_yeast_ in terms of both topology and age-dependency. In all three models, the network starts from a small number, N_0_ = 4 of seed nodes and a new node is added until the total number of nodes reaches N = 3,000, which is comparable to the PPI_yeast_ (LC) with 3,268 nodes and 12,058 edges. In the PA and AP models, a fixed number of edges (ΔE = 4) are added for each new node, which makes the final network size similar to the PPI_yeast_. The link probability (P) is proportional to the degree in the PA model (P ∼ k) and inversely proportional in the AP model (P ∼ k^−1^). For the DD model, we employ one of the simplest models by Vázquez *et al.* [12]: One node (i) is duplicated randomly, the new node (i') is connected to all of the neighbors of i, and then the duplicates (i and i') are linked with a small probability p. For each neighbor (j) of the duplicates, one of the two links (i,j and i',j) is chosen randomly and deleted with the divergence probability q. Because this model may generate orphan nodes that are not connected to any other nodes, orphan nodes were removed in each duplication step.

Surprisingly, none of the three models satisfied all of the characteristics of PPI_yeast_ (the 2nd, 3rd and 4th columns in Figure 2 for the PA, DD and AP model respectively). The PA and DD models generate scale-free networks and show degree-dissortativity and the DD model also shows some degree of hierarchical modularity. However, both the PA and DD models show an inverse interaction density pattern with negative ΔD. In contrast, although the AP model shows positive ΔD similar to PPI_yeast_, it deviates greatly in terms of topological characteristics. That is, the PPI_yeast_ seem to show mixed characteristics, with the network topology resembling that of the DD (PA) model but with the interaction density similar to the AP model. Also, all three models generally show much lower levels of modularity than the PPI_yeast_ (the row II in Figure 2). We further examined two more variants of DD models, where the divergence of edges between the duplicates is asymmetric (DD_asym_) by Ispolatov et al. [14] and allow rewiring as well as asymmetric (DD_asym-rw_) by Pastor-Satorras et al. [11]. None of the tested DD variants were in good agreement with PPI_yeast_, showing negative ΔD and lower clustering coefficient. In yeast, whole genome duplication (WGD) occurred relatively recently after speciation of *Kluyveromyces waltii* and *Saccharomyces cerevisiae* [31]. Simulation of WGD at the last stage of DD model did not improve the model either (data not shown). As a global topological index, the shortest path length was also examined but provided little discrimination among the tested models due to high variability depending on model parameters (DD model) and the choice of yeast PPI data set. Each model was simulated 100 times and the summary of the network properties is given in Table S2.

While additional variants of each model might be considered [13],[20],[32], the critical characteristics of each model are largely captured by these canonical models, e.g. the DD model has no mechanism to generate positive ΔD. The inconsistency of these models with the interaction age density of real PPI networks clearly suggest that none of these canonical models is sufficient in itself to qualify as a valid model for the evolution of the yeast PPI network.

### A Crystal Growth Model

**Figure 3:**
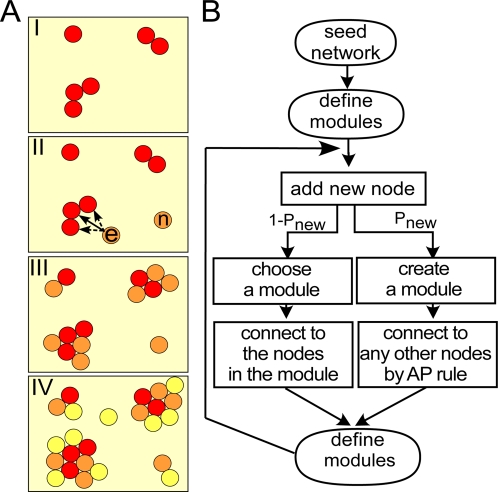
A schematic diagram (A) and a flowchart (B) show the process of network growth by the CG model. (A) The CG model mimics sequential incorporation of new proteins to crystals grown in solution. In stage I, the initial set of proteins (red) form seeds of new crystals. In stage II, a new protein is added, which either forms a new seed crystal (n) or attaches to an existing crystal (e). In the latter case, the protein e attaches to one protein in the crystal (solid arrow) and then further interacts with nearby proteins (dotted arrow). In stages III and IV, the second- (orange) and third- (yellow) generation proteins repeat the process of stage II, with the result that the early generation tends to be located at the core of each crystal and the late generation at the periphery. (B) Similarly, the CG model starts with a small number of seed nodes (N_0_). In each cycle, modules are defined and a new node is added that makes a fixed number of connections (ΔE). A new node creates a new module at a probability P_new_ and makes connections to any other node in accordance with the AP rule. Otherwise, one module (crystal) is randomly selected and the new node is connected exclusively to the nodes in the selected module. After ΔE connections are made, modules are redefined and the cycle is repeated.

To better address both topological and age properties of real networks, we developed an alternative model for PPI network evolution called the crystal growth model (CG), in which we view the growth of a PPI network as analogous to incorporating new proteins into crystals grown in solution (Figure 3A). The two key ideas are as follows. First, the connection probability increases with the availability of unoccupied surface, and thus the model follows anti-preferential attachment rule (AP rule). Second, the connections of a new node tend to be limited within a network module, as observed in growing crystals and here termed as *localized connection*.

The procedure of the CG model is illustrated in Figure 3B. As in the PA and AP models, the CG model starts with a few seed nodes (N_0_ = 4), and a new node makes a fixed number of connections (here, ΔE = 4) to existing nodes. For each new node added, network modules are redefined as local dense regions in the network. As modules emerge as a result of network growth and are not pre-defined artificially, the number of modules (M) is not fixed but may increase or decrease in each step. With a small probability P_new,_ a new node becomes a new module by itself and makes connections ΔE times to other nodes in accordance with the AP rule. Otherwise, an existing module is selected randomly, and the new node is committed to the module by making connections exclusively within the selected module. The connection takes two steps, dubbed “*anchoring and extension*”. In the anchoring step, the new node connects to an anchor node in the module in accordance with the AP rule, and then, in the extension step, the new node further connects only to the neighbors of the anchor node in the module. Connections are created randomly to neighboring nodes until ΔE connections are made. The anchoring and extension steps are analogous to the node *e* in Figure 3A (stage II). Therefore, the CG model is inherently highly module-oriented. In case that the neighbors of the anchor node are fewer than ΔE in the chosen module, the module selection and connection step is repeated until ΔE connections are made and the new node becomes connected to multiple modules.

The CG model introduces two parameters, how to define the network modules and how frequently a new module is created (P_new_). A network module is generally defined as a densely connected sub-network, and there are various ways to partition a network into modules. Most stringently, modules can be defined as complete subgraphs or cliques, and more loosely they can be defined as k-cores, triangularly connected components (TCC) and so on. We tested two different module definitions, one by Newman [33] and the other by TCC. We mainly discuss the results by the Newman definition, but results using TCC were highly similar (Figure S3). Also, P_new_ was assigned as M^−1^ because the chance of creating a new module generally decreases with the number of existing modules (M). Setting a small, fixed value of P_new_ also show a similar result (data not shown).

Networks generated by the CG model show a remarkable similarity to real PPI networks for all tested network properties. A typical result of the CG model is shown in the 5th column in Figure 2. The topology of the CG model shows a scale-free, a hierarchical modular, and a degree-dissortative characteristic. Interestingly, both the magnitude and the shape of clustering coefficient was similar to the PPI_yeast_ in the C(k) ∼ k plot (the row II in Figure 2). The CG model also shows a similar pattern of degree-dissortativity and interaction density with a positive ΔD (the row III and IV in Figure 2). These characteristics were robust with varying network sizes, e.g., N = 1,000 and N = 5,000 (data not shown).

### Comparison of the Network Properties between Network Growth Models and Yeast PPI Network

**Figure 4:**
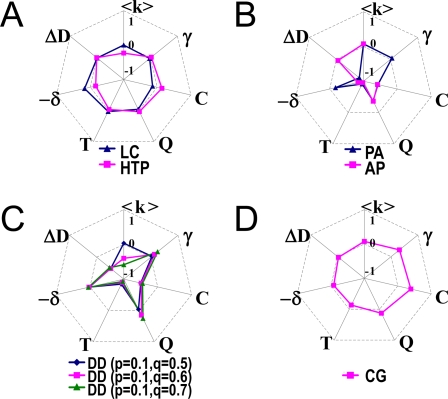
The comparison of network property indices between the yeast PPI networks and the models tested. (A) PPI_yeast_, (B) the PA and AP models, (C) the DD model at p = 0.1, q = 0.5∼0.7, and (D) the CG model. In (B), the scale-free index, γ, of the AP model is not shown because the resulting network is not scale-free. The properties of the CG model are more similar to PPI_yeast_ than those of the PA, AP, and DD models. Index values are normalized so that the average indexes of LC and HTP are zero, calculated as I_norm_ = (I_raw_−I_yeast_)/I_range_, where I_norm_ is the normalized index and I_raw_ is the index value of each model. I_yeast_ is the average index between LC and HTP except for <k>, where I_yeast_ is set to the average degree of LC because <k>_LC_ is similar to <k> = 8.0 in the PA, AP, and CG models. The denominator I_range_ is set to max(I_raw_) observed in LC, HTP, and the models, except for δ and ΔD showing both negative and positive values. In the case of ΔD and –δ, the denominator I_range_ is set to max(I_raw_)−min(I_raw_) because these indexes range from negative to positive values in LC, HTP, and the models. The sign of δ is reversed to −δ to give the index positive values for LC and HTP.

The canonical models were shown to significantly deviate from the PPI_yeast_, but the CG model shows a good agreement not only qualitatively but also quantitatively (Figure 4). For objective comparison of the models, various indices were used to summarize the network characteristics, including power-law degree distribution (γ), hierarchical modularity (Q, C, C(k) ∼ k curve shape and triangle density, T), dissortativity (δ), and the age-dependency of interaction density (ΔD).

DD and PA show an inverse age-dependency of PPI_yeast_ and much less modularity in terms of clustering coefficient and triangle density although they show scale-free degree distributions (Figure 4B and 4C). The AP model was not able to generate a scale-free network and significantly deviates from the PPI_yeast_ for all the network indices tested except ΔD (Figure 4B). Only the CG model was comparable to the PPI_yeast_ in terms of all the network indices tested, including both scale-freeness (γ) and age-dependency (ΔD) (Figure 4D). In particular, only the CG model shows an extremely high degree of modularity comparable to the PPI_yeast_ in terms of both clustering coefficient and triangle density due to its inherently module-oriented mechanism. The mixing exponent (δ) is intermediate between LC and HTP. Therefore, of all models considered, the CG model agrees best with both topological and age-dependencies of the actual yeast PPI network. In Table S2, the network property indices are summarized for all the models tested after 100 simulations of each model.

### Age-Dependency of Homodimeric Frequency in CG Model

**Figure 5:**
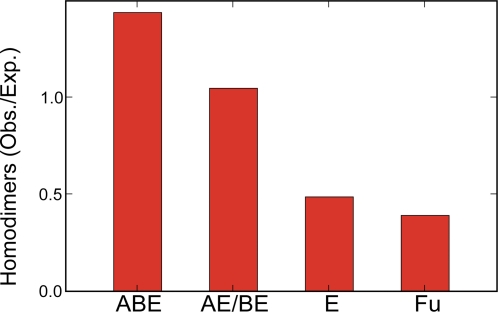
The frequencies of homodimers are age-dependent. The ratio between the observed (Obs.) and the expected (Exp.) number of homodimers is plotted for each age group, calculating for each age group the fraction of homodimeric proteins divided by the fraction of total yeast proteins accounted for by that age group.

In the CG model, homodimers would be more frequent in older groups because there are simply fewer proteins with which to make connections in earlier stages. The age distribution of homodimeric interactions was exactly in the order of ABE>AE/BE>E>Fu among the 166 homodimeric yeast proteins collected from UniProt [34] and the literature (Figure 5, Dataset S4). This result is also consistent with previous studies from protein 3-D structures, in which ancient proteins were shown to be highly enriched with homodimeric or paralogous interactions [35],[36]. Although the PA and AP would also generate a similar trend, the resulting topology and/or interaction density greatly deviate from PPI_yeast_ to be considered as a realistic model. In the DD model, a fixed interaction probability, *p* is set for interactions between duplicates (paralogs), therefore implicitly predicts homodimeric formation is age-independent because most paralogous interactions originate from homodimeric interactions and were not created *de novo* [37],[38]. Thus, the age-dependency of homodimeric frequencies is a good support for the CG model, which has not previously been applied as a criterion for valid network evolution models.

### Sub-Networks and Spatial Arrangement of Complex Subunits

Within the sub-networks of known complexes from MIPS, protein subunits tend to be either more likely to be connected among similar age groups in agreement with the general tendency of positive ΔD in the full yeast PPI networks (Figures S4A and S4B) or consist mostly of the same age group, reflecting the creation of a new protein module at a certain evolutionary lineage e.g. actin-associated proteins (Figure S4E). Other complexes form densely connected sub-networks, where age-dependency was not evident, e.g. RNA polymerase I and III (Figures S4C and S4D).

We further validated the CG model by inspecting the 3-D subunit arrangement of protein complexes according to age. Obviously, a protein subunit of a stable complex interacts mostly with the subunits of its participating complex. When a subunit is in contact with multiple other subunits in a protein complex, it is most likely that the partner subunits are spatially close, often interacting among themselves as well. For transient interactions, the member proteins can interact with fewer spatial constraints but the interactions are much denser within each biological module, e.g. as for a MAP kinase signaling pathway or transcription initiation complex. Therefore, a protein tends to interact in a highly “localized” manner within the biological modules it belongs to. None of the canonical models has such a module-oriented mechanism as the CG model. In the CG model, older subunits of protein complexes would tend to be more centrally located than younger ones because each protein is attached in the order of its age. Therefore, it is more likely that older subunits are aggregated centrally and younger subunits are scattered at the periphery in a protein complex.

To examine this trend among known protein complexes, we collected protein complexes from the Protein Databank (PDB) which consisted of at least 3 protein chains, with at least 2 age groups represented; these are stringent criteria that strongly limit the number of available complexes. After removing inappropriate complexes, such as non-protein structures, viral proteins, antibodies and small peptides, a non-redundant set of 12 multi-protein complexes was collected that met these criteria (detailed descriptions are in Methods).

**Figure 6:**
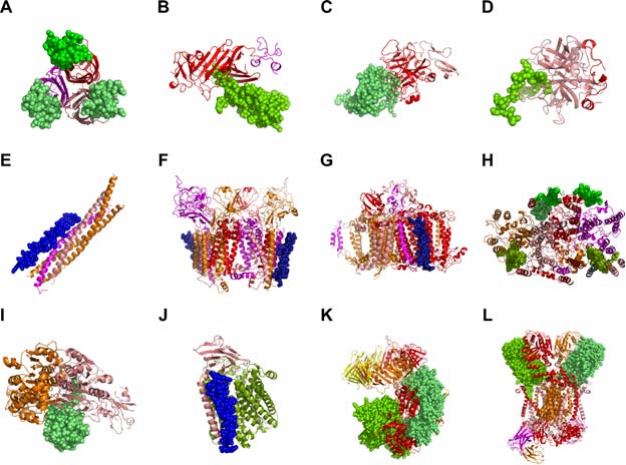
The spatial subunit arrangement of known multi-protein complexes is consistent with the CG model. Subunits of all 12 known multi-protein complexes with at least three proteins and two-age groups are colored according to their age groups: The most ancient group, ABE, is colored in red tones (yellow, pink, magenta, orange, red). The AB, AE, or BE groups are in green tone, and the most recent A, B, and E groups are in blue. For visual clarity, the older group(s) is presented in cartoon models and the youngest group in space-filling models in each complex. The age group assignments in (K) and (L) were ambiguous because the chains assigned AE could be assigned to ABE if the BLAST hit cut-off was slightly relaxed to 25% instead of 30% sequence identity (the “twilight zone” for homology detection). Therefore, (K) and (L) may, in fact, consist of the subunits of the ABE group only. The subunits are in various configurations. In (A) and (L), the younger subunits are spatially separated, but the older subunits are aggregated. In (B–E), two old subunits (three in (E)) and one young subunit are linearly connected. In all four cases, the older subunits are all connected without insertion of the younger subunit in the middle. In (F–H), the subunits form a trans-membrane helix bundle, where young subunits are always located at the periphery while old subunits are at the center. In (I) and (J), all the subunits are in contact with each other. In the case of (K), there are two modules—the clamp (upper homo-trimeric ring) and the clamp loader (lower hetero-pentameric ring). Considering the clamp loader alone, both younger and older subunits are separated. (A) APRIL and TACI (TNF receptor) complex (Protein databank code 1xu2). (B) Urokinase receptor, urokinase, and vitronectin complex (3bt1). (C) Factor Xa/NAP5 complex (2p3f). (D) Thrombin-PAR4 complex (2pv9). (E) Complexin/SNARE complex. (F) Cytochrome b6f complex (2e76). (G) Cyanobacterial photosystem I (1jb0). (H) Photosynthetic Oxygen-Evolving Center. A cross-section of the trans-membrane helix bundle is shown (1s5l). (I) APPBP1-UBA3-NEDD8 complex (1r4m). (J) Cytochrome ba3 Oxidase (1xme). (K) DNA clamp–clamp loader complex (1sxj). (L) Cythochrome bc complex (1ezv).

In general, older subunits tend to be aggregated centrally (red tone), while younger ones are separated peripherally (green and blue) (Figure 6). In Figure 6A, older subunits form trimeric aggregates but younger ones were separated. There were four linear complexes and no younger subunit intervened between the older ones (Figure 6B–6E). That is, the contacts were always in e.g. the ABE-ABE-AE configuration but not the ABE-AE-ABE, as predicted by the CG model, in which ABE-ABE is connected first and ABE-AE later. The other three complexes contain trans-membrane helix bundles, where the younger helix chain is located at the periphery (Figure 6F–6H). Of the remaining four complexes, two had all subunits contacting each other and were thus non-informative (Figure 6I–6J), and two had ambiguous age assignments for subunits, although the putatively younger subunits were spatially separated (Figure 6K–6L). Considering the eight informative complexes (Figure 6A–6H), the observed subunit arrangements significantly support the CG model at P = 0.019, based on random permutations of chain arrangements within the asymmetric unit of each complex.

It is notable that the total degree of PPI_yeast_ is underestimated relative to the actual degree due to homomeric interactions and subunit stoichiometry. For example, the APRIL-TACI complex (Figure 6A) was the form A_3_B_3_ with the degree k_A_ = 3 (two homomeric, one heteromeric) and k_B_ = 1 (one heteromeric). In contrast, only one interaction (A–B) would be counted for each subunit in PPI_yeast_.

## Discussion

The validity of network evolution models have been measured mainly by the resulting network topology, such as a power-law degree distribution, hierarchical modularity and dissortativity as observed in real PPI networks. Accordingly, the DD model has been thought of as the principal mechanism for PPI network evolution. Here, we dissect the history of PPI network evolution by inspecting several protein age-dependent patterns such as interaction density, homodimeric frequency, and the 3-D spatial arrangement of subunits within multiprotein complexes. The age-dependencies are shown to be very effective in discriminating the validity of different models as summarized in Table 1. The tested aspects of age-dependency were independent of topologies as well as of each other, and are thus highly useful as orthogonal criteria for valid models. Importantly, the age-dependent interaction patterns provided insights on PPI evolution, suggesting evidence against the DD model as the dominant mode of PPI network evolution, instead supporting an alternative model, the CG model.

**Table 1:** Properties of yeast PPI networks and the tested network evolution models.

		PPI	PA	DD*	AP	CG
Scale-free		Yes	Yes	Yes	No	Yes
Modularity		Yes	No	Yes	No	Yes
	Q	High	Low	High	Low	High
	C(k)	High	Low	Medium	Low	High
	C(k) ∼ k shape	-	Different	Similar	Different	Similar
	Triangle density	High	Low	Low∼Medium**	Low	High
Dissortativity (δ)		Yes	Yes	Yes/No**	No	Yes
Age-dependency						
	Interaction density (ΔD)	Yes	No	No	Yes	Yes
	Homodimeric frequency	Yes	Yes	No	Yes	Yes
	3-D subunit arrangement in protein complexes	-	Not explicitly modeled	Non-supportive	Not explicitly modeled	Supportive

*: Results for the DD model were collected at typical values (p = 0.1, q = 0.6). Results for scale-freeness and age-dependency are robust to changes in these parameters. However, aspects of modularity and dissortativity of the DD model vary with these parameters and the specific choice of DD model, indicated with ^**^.

In the CG model, we view the PPI network as sparse and dynamic protein crystals *per se*. The CG model mimics the process of growing protein crystals in solution by sequentially adding each protein. Despite the huge differences in time scale and heterogeneous composition, PPI network evolution likely obeys similar constraints on growing protein crystals. In the CG model, a protein complex or a tightly linked module is analogous to individual crystals, and the number and membership of modules are not pre-defined but rather emerge naturally in each growing step. Crystals grow around multiple nuclei just as protein networks consist of multiple modules/complexes. New modules are generated as the genome size increases and novel function evolves in higher organisms, in a manner similar to how a new crystal forms occasionally through new nucleation events.

The CG model exploits two keys ideas, the first being that the chance of new connection is proportional to the availability of free surface, which is a feature readily recognized by a new protein molecule; this results in an anti-preferential attachment (AP) rule. Although the same surface of a protein can be involved in multiple interactions with different partners through spatial and temporal differentiation, such a factor uniformly increases the capacity of interactions in any protein. Therefore, the connection probability is still positively correlated with the available surface area. These results agree with those of Kim et al. [39], which show that the evolutionary rate is anti-correlated with available surface area. There, multi-interface hubs were nearly four times more frequent than single-interface hubs, reflecting the dominant connection mode of the AP rule. The second key idea is that once an initial connection is made, the subsequent connections are localized to the neighbors of the initial partner within the same module. This localized connection enforces high modularity, similar to that observed in real PPI networks.

At the basis of the crystal growth model is the notion that new interactions form preferentially within existing physical complexes (enforcing modularity), and thus are limited by available protein surface area (the AP rule). Thus modularity & the AP rule both arise due to simple physical constraints of which proteins are most accessible to each other. Recently, Levy and colleagues has shown that the successive steps of homo-oligomeric assembly mimics the evolutionary pathway [38]. The CG model expands this idea, where crystal growth reproduces the evolution of the entire PPI network.

Given that the CG model follows an AP rule, how does it generate scale-freeness or “the rich get richer” connectivity? In the CG model, the network grows by *anchoring and extension*, where a node increases its degree either by becoming an anchor node (anchoring) or by being the neighbor of the anchor node (extension). Therefore, the highly connected nodes have greater chances to increase their degree within each module because they have more opportunities to have anchors as their neighbors. Therefore, the CG model implicitly implements the preferential attachment (PA) rule within each module in a manner similar to the DD model, where the nodes increase their degree by having duplicating genes as their neighbors.

Our result suggests that the CG model is a more plausible mechanism for PPI network evolution than the DD model. First, all the age-dependent aspects tested agree well with the CG model but disagree with the DD model. Second, the CG model is more comprehensive than the DD model in that the CG model can accommodate both gene duplication and horizontal gene transfer as the origins of new nodes (genes). Practically, the DD model may be applicable only to ∼20% of the yeast proteome having identifiable duplicates [40]. The CG model also embodies the rapid divergence of gene duplicates [17] by the AP rule, which avoids competition for the same interface on common partners and connects to new partners with less occupied surfaces. Finally, the CG model is more robust than the DD model. The DD model shows a highly variable degree distribution depending upon parameters and network sizes [14],[41]. In contrast, the CG model shows stable characteristics regardless of network size or different module definition methods. Taken together, these strongly suggest that the DD model is unlikely to be the principal, and strongly unlikely to be the sole, mechanism of PPI network evolution.

The age-dependency of interaction density also sheds light on a more fundamental question regarding the mechanism of PPI network evolution. It has been hypothesized that inherent features of proteins, such as stickiness and hydrophobicity are dominant factors in shaping the global network structure [6]. However, the observed age-dependency is inconsistent with such a hypothesis and suggests that a stochastic process played a major role. For example, the yeast PPI network shows the patterns of both D_ABE,AE/BE_>D_ABE,E_ and D_AE/BE,Fu_<D_E,Fu_ (the row IV in Figure 2). The connection probability cannot depend solely upon a feature such as protein length or surface hydrophobicity because no single feature (F) can satisfy F_AE/BE_>F_E_ (with common F_ABE_) and F_AE/BE_<F_E_ (with common F_Fu_) simultaneously.

Power-law distributions have been commonly observed in various types of networks, such as the Internet, social networks, and biological networks. However, the growth of a PPI network poses unique constraints compared to other types of networks. For example, in an airline or railroad network, each new connection is made by considering the context of global network topology (e.g., to minimize average path length), which seems intuitively unlikely to be the case in PPI networks. The CG model follows two simple constraints of available free surface and localized connection, which are physically plausible and depend only on local context but not global topology. With these minimal assumptions analogous to growing protein crystals, the CG model recapitulates remarkably well the age-dependencies as well as the network topologies of the yeast PPI networks.

## Methods

### Yeast Protein Interaction Data

Two independent sets of yeast protein-protein interaction data were collected using a method essentially identical to that described by Batada et al. [23],[24], only differing in that the HTP set was collected from the original publications instead of from BioGrid [42]. We compiled the HTP set from Uetz *et al.* [43], Ito *et al.* [44], the merged set of Gavin *et al.* [45],[46], Ho *et al.* [47], and Krogan *et al.* [48], and then filtered out the interactions supported by only a single experiment. Repeated and reciprocal assays were considered as independent experiments even if they were performed in the same publication. The LC data set was collected from the latest release of BioGrid, excluding high-throughput data. Ribosomal proteins were removed from both LC and HTP data sets. All protein-RNA interactions and interactions supported only by co-localization or co-fractionation were removed. We further removed interactions supported only by Ptacek *et al*. [49], Grandi [50], Collins *et al*. [51], or Fields *et al*. [52].

### Yeast Protein Age Groups

Pfam domains were assigned for yeast proteins using BioMart (http://www.biomart.org). The taxonomic distributions of Pfam domains were obtained for archaea (A), bacteria (B), eukaryotes (E), and fungi (F) (http://www.sanger.ac.uk/Software/Pfam). According to these distributions, each Pfam domain was assigned to one of the age groups ABE, AE/BE, E, and F. The group ABE includes the oldest proteins common to all three kingdoms, while group F is the youngest, being specific to fungi. As yeast is a eukaryote, groups A, B, and AB do not occur. A protein's age group was assigned as the youngest age of its constituent Pfam domains—e.g., E for a protein with domains from ABE and E (Dataset S3, Figure S1).

### Interaction Density and ΔD

Interaction density D_m,n_ measures the normalized interaction density between two age groups m, n (m<n). ΔD measures the interaction preference of a new node by the age differences. A positive value of ΔD indicates that a new node makes connections more frequently with close age groups than with distant ones.

First, the normalized interaction density D_m,n_ between two age groups m,n (m<n) is calculated as
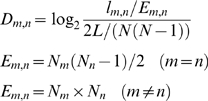
where l_m,n_ is the number of edges between the two age groups m and n, and E_m,n_ is the number of all possible interactions between the two groups. N_m_ and N_n_ are the number of nodes in the age groups m and n, respectively, L is the total number of edges, and N is the total number of nodes in the network. Then the average interaction density gradient, ΔD, of a network is defined as
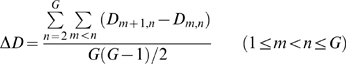
where G (G≥2) is the number of age groups.

### Measure of Modularity

The modularity of a network is measured by the modularity index Q by Newman [29] after its modules are defined using the method described in [33]:
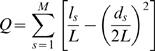
where M = the total number of modules, L = the number of total edges in the network, l_s_ = the number of edges within the module s, and d_s_ = the sum of the degrees of the module s. The modularity index Q measures the difference between the intra-module interaction density and the expected interaction density at random for a given partition, where Q≈0 for a random network and Q = 1 for a completely modular network [53].

### Protein 3-D Complexes Data

The list of PDB entries and 3-D coordinates were obtained from PQS (Protein Quaternary Structure Server, ftp://ftp.ebi.ac.uk/pub/databases/msd/pqs). First, we took the PDB entries having three or more protein chains. The PDB entries annotated as crystal packing interfaces by PQS or from non X-ray crystallographic method were excluded.

The protein chain clusters at 30% sequence identity cut-off were downloaded from PDB (Protein Data Bank, ftp://ftp.wwpdb.org). PDB entries consisting of the same set of NR30 clusters were grouped together regardless of the number of chains and one representative PDB entry was selected in each group as NR30 entries.

For NR30 entries, the age group of each PDB chain was assigned using BLAST against NR90 set of archaea, bacteria and eukaryote sequences from UNIPROT (ftp://ftp.uniprot.org/pub/databases/uniprot) using >30% identity and >30 alignment length as criteria. We took only the PDB entries consisting of two or more protein age groups and further applied a number of filters manually, excluding the entries with DNAs, RNAs, viral proteins, small peptides (<30 amino acids) and immunoproteins such as antibodies and MHCs with antigens. Where available, ambiguous quaternary structures were removed by comparing the data from PQS, PDB biological units and 3D complex databases [54].

## Supporting Information

Dataset S1 LC dataset
(0.20 MB TXT)

Dataset S2 HTP dataset
(0.11 MB TDS)

Dataset S3 The age group assignment of yeast genes
(0.08 MB TDS)

Dataset S4 The list of homodimeric proteins and their age group assignment
(0.01 MB TDS)

Figure S1 The protein ratio of different age groups in yeast PPI networks. LC: literature-curated, HTP: high-throughput, LC+HTP: the union of LC and HTP.
(0.08 MB PDF)

Figure S2 The network properties of the HTP, LC+HTP, and Y2H-union dataset. The plots in each row, I-IV, indicate (I) The degree distribution P(k), (II) the clustering coefficient C(k), (III) the average degree of nearest neighbors <k_nn_>(k), and (IV) the interaction density pattern (ΔD) between protein age groups. HTP, LC+HTP, and Y2H-union set show similar characteristics as LC dataset.
(0.29 MB PDF)

Figure S3 The network properties by the CG model, where the network modules were defined by TCC (triangularly connected components) instead of the Newman's method. The network structure is still similar to the yeast PPI networks, showing scale-free, hierarchical modular, degree-dissortative characteristics and an interaction density pattern of DD>0. (A) The degree distribution P(k), (B) the clustering coefficient C(k), (C) the average degree of nearest neighbors <knn>(k), (D) the interaction density pattern between protein age groups.
(0.09 MB PDF)

Figure S4 Age-dependent interaction patterns of several MIPS complexes in the LC+HTP set. In mRNA splicing (A) and replication (B) complexes, the subunits of the same age group are more likely to be connected. In RNA polymerase I & III (C and D), most subunits are densely connected to each other, therefore age-dependency is not evident. In the case of actin-associated proteins, most subunits are of the same age group (E), reflecting a relatively recently emerged module.
(0.52 MB PDF)

Table S1 The network characteristics of the yeast PPI data.
(0.06 MB PDF)

Table S2 The network characteristics of the network growth models
(0.13 MB PDF)
