# Supplementary figures and images for: Age-Dependent Evolution of the Yeast Protein Interaction Network Suggests a Limited Role of Gene Duplication and Divergence
Source: PLoS Comput Biol. 2008 Nov 28;4(11):e1000232. doi: 10.1371/journal.pcbi.1000232 (PMC2583957; doi:10.1371/journal.pcbi.1000232)

# LC

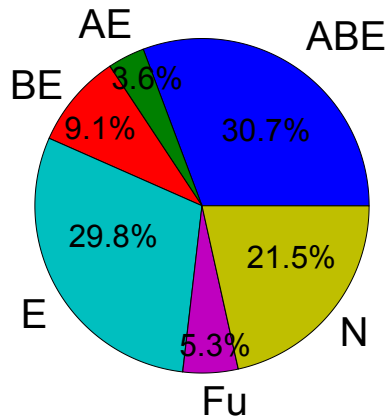

# HTP

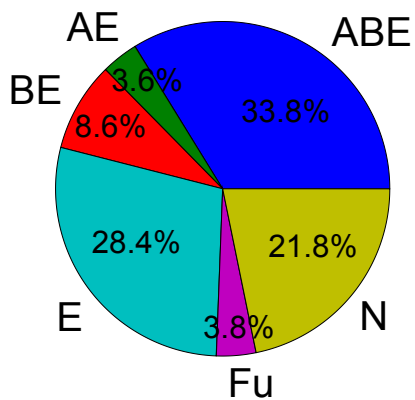

# LC+HTP

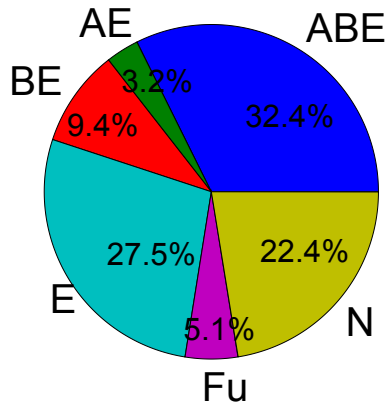

Supplement: Figure S1 — The protein ratio of different age groups in yeast PPI networks. LC: literature-curated, HTP: high-throughput, LC+HTP: the union of LC and HTP. (0.08 MB PDF) [file pcbi.1000232.s005.pdf]

HTP

LC+HTP

Y2H-union

I

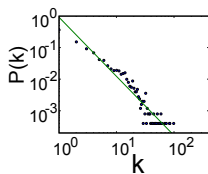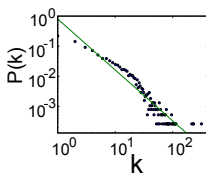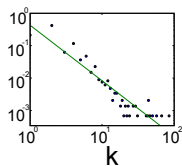

II

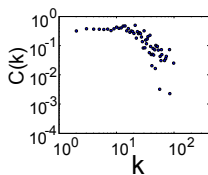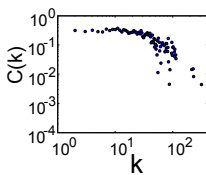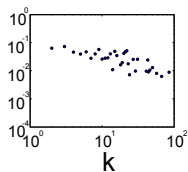

III

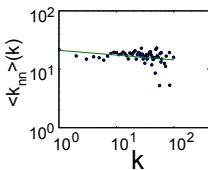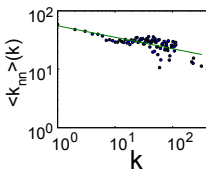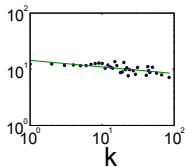

IV

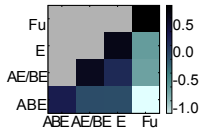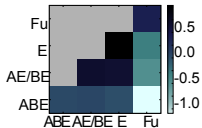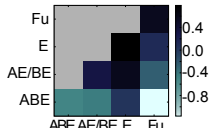

Supplement: Figure S2 — The network properties of the HTP, LC+HTP, and Y2H-union dataset. The plots in each row, I-IV, indicate (I) The degree distribution P(k), (II) the clustering coefficient C(k), (III) the average degree of nearest neighbors (k), and (IV) the interaction density pattern (ΔD) between protein age groups. HTP, LC+HTP, and Y2H-union set show similar characteristics as LC dataset. (0.29 MB PDF) [file pcbi.1000232.s006.pdf]

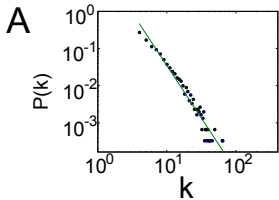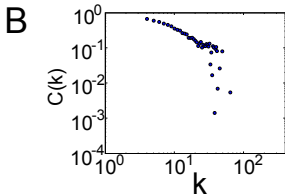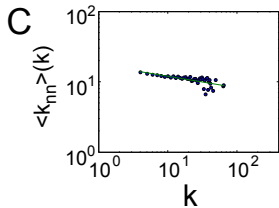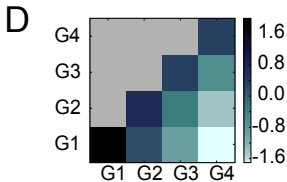

Supplement: Figure S3 — The network properties by the CG model, where the network modules were defined by TCC (triangularly connected components) instead of the Newman's method. The network structure is still similar to the yeast PPI networks, showing scale-free, hierarchical modular, degree-dissortative characteristics and an interaction density pattern of DD>0. (A) The degree distribution P(k), (B) the clustering coefficient C(k), (C) the average degree of nearest neighbors (k), (D) the interaction density pattern between protein age groups. (0.09 MB PDF) [file pcbi.1000232.s007.pdf]
