# Supplementary material for: Age-Dependent Evolution of the Yeast Protein Interaction Network Suggests a Limited Role of Gene Duplication and Divergence
Source: PLoS Comput Biol. 2008 Nov 28;4(11):e1000232. doi: 10.1371/journal.pcbi.1000232 (PMC2583957; doi:10.1371/journal.pcbi.1000232)
Supplement: Figure S4 — Age-dependent interaction patterns of several MIPS complexes in the LC+HTP set. In mRNA splicing (A) and replication (B) complexes, the subunits of the same age group are more likely to be connected. In RNA polymerase I & III (C and D), most subunits are densely connected to each other, therefore age-dependency is not evident. In the case of actin-associated proteins, most subunits are of the same age group (E), reflecting a relatively recently emerged module. (0.52 MB PDF) [file pcbi.1000232.s008.pdf]

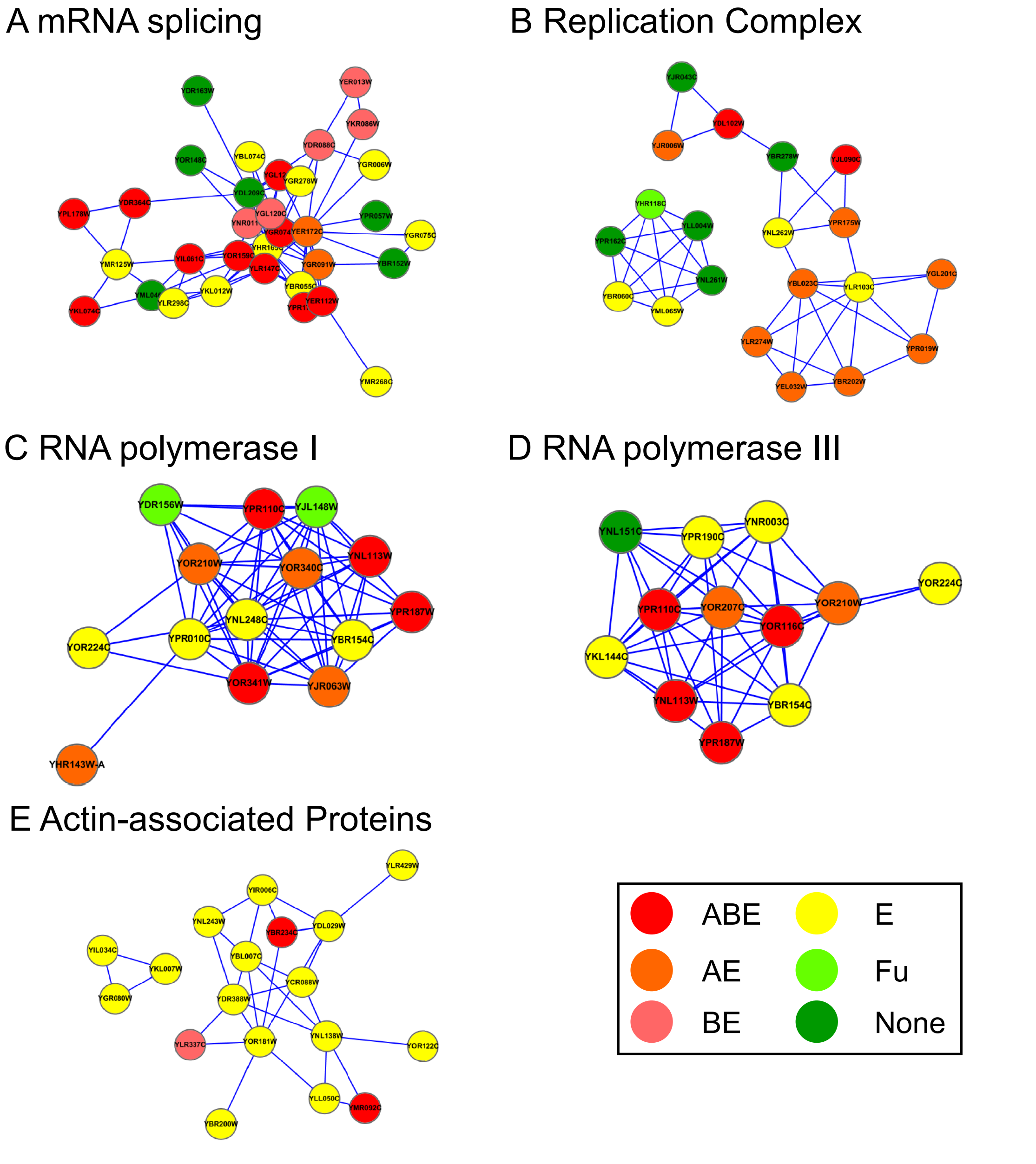
A mRNA splicing
YDR163W
YER013W
YKR086W
YDR088C
YOR148C
YBL074C
YGR278W
YGR006W
YDL209C
YPL178W
YDR364C
YGL120C
YPR057W
YER172C
YGR075C
YIL061C
YOR159C
YMR125W
YLR147C
YGR091W
YBR152W
YBR055C
YER112W
YLR298C
YKL012W
YKL074C
YMR268C
B Replication Complex
YJR043C
YDL102W
YJR006W
YBR278W
YJL090C
YHR118C
YLL004W
YPR162C
YNL262W
YPR175W
YNL261W
YBR060C
YML065W
YBL023C
YLR103C
YGL201C
YLR274W
YPR019W
YEL032W
YBR202W
C RNA polymerase I
YDR156W
YPR110C
YJL148W
YOR210W
YOR340C
YNL113W
YPR187W
YNL248C
YOR224C
YPR010C
YBR154C
YOR341W
YJR063W
YHR143W-A
D RNA polymerase III
YNL151C
YPR190C
YNR003C
YOR224C
YPR110C
YOR207C
YOR210W
YOR116C
YKL144C
YNL113W
YBR154C
YPR187W
E Actin-associated Proteins
YLR429W
YIR006C
YNL243W
YBR234C
YDL029W
YIL034C
YBL007C
YKL007W
YCR088W
YGR080W
YDR388W
YLR337C
YOR181W
YNL138W
YOR122C
YLL050C
YMR092C
YBR200W
ABE
E
AE
Fu
BE
None
