# Supplementary material for: Age-Dependent Evolution of the Yeast Protein Interaction Network Suggests a Limited Role of Gene Duplication and Divergence
Source: PLoS Comput Biol. 2008 Nov 28;4(11):e1000232. doi: 10.1371/journal.pcbi.1000232 (PMC2583957; doi:10.1371/journal.pcbi.1000232)
Supplement: Table S1 — The network characteristics of the yeast PPI data. (0.06 MB PDF) [file pcbi.1000232.s009.pdf]

Table S1. The network characteristics of the yeast PPI data

	LC		HTP		HTP_LC	
	Total	[1]Giant	Total	Giant	Total	Giant
Number of nodes	3268	3186	2488	2263	3780	3721
Number of edges	12058	12004	6766	6617	16505	16471
[2]$\langle k \rangle$	7.4	7.5	5.4	5.8	8.7	8.8
[3]γ	-1.7	-1.7	-1.9	-1.9	-1.7	-1.7
Clustering Coefficient (C)	0.13	0.13	0.21	0.21	0.14	0.14
Modularity (Q)	0.62	0.62	0.69	0.68	0.55	0.55
[4]Triangle density (T)	1.2	1.2	1.1	1.2	1.4	1.4
[5]δ	-0.30	-0.31	-0.08	-0.10	-0.20	-0.20
ΔD	0.5458	0.5318	0.5689	0.5495	0.5207	0.5062

[1] The largest connected component
[2] average degree
[3] the degree exponent in $P(k) \sim k^{-\gamma}$
[4] the number of triangles per edge
[5] the mixing exponent in $\langle k_{nn} \rangle(k) \sim k^{-\delta}$
