# Supplementary material for: Age-Dependent Evolution of the Yeast Protein Interaction Network Suggests a Limited Role of Gene Duplication and Divergence
Source: PLoS Comput Biol. 2008 Nov 28;4(11):e1000232. doi: 10.1371/journal.pcbi.1000232 (PMC2583957; doi:10.1371/journal.pcbi.1000232)
Supplement: Table S2 — The network characteristics of the network growth models (0.13 MB PDF) [file pcbi.1000232.s010.pdf]

Table S2. The network characteristics of the network growth models

Model	[1]parameters		[2]<k>	[3]γ	Clustering Coefficient (C)	Modularity Index (Q)	[4]Triangle density (T)	[5]δ	ΔD
PA	-		8.0±0.0	-2.0±0.1	0.010±0.0005	0.33±0.003	0.066±0.005	-0.08±0.03	-0.80±0.03
DD	p=0.05	q=0.5	7.4±0.8	-1.9±0.07	0.015±0.002	0.62±0.05	0.080±0.01	-0.27±0.07	-0.28±0.04
		q=0.6	3.9±0.2	-2.2±0.08	0.019±0.003	0.79±0.03	0.046±0.007	-0.26±0.07	-0.27±0.04
		q=0.7	2.4±0.08	-2.6±0.1	0.024±0.004	0.88±0.01	0.027±0.004	-0.27±0.06	-0.22±0.04
	p=0.1	q=0.5	7.7±0.9	-1.9±0.07	0.030±0.004	0.62±0.06	0.16±0.02	-0.20±0.06	-0.27±0.02
		q=0.6	4.0±0.3	-2.2±0.08	0.035±0.004	0.79±0.04	0.087±0.008	-0.21±0.07	-0.27±0.05
		q=0.7	2.5±0.08	-2.6±0.1	0.045±0.006	0.88±0.01	0.053±0.005	-0.22±0.06	-0.21±0.05
	p=0.2	q=0.5	8.3±0.8	-1.9±0.06	0.055±0.006	0.61±0.05	0.30±0.018	-0.13±0.05	-0.28±0.04
		q=0.6	4.3±0.3	-2.2±0.08	0.070±0.008	0.79±0.04	0.17±0.01	-0.13±0.05	-0.24±0.04
		q=0.7	2.7±0.09	-2.6±0.1	0.085±0.007	0.88±0.01	0.099±0.007	-0.14±0.05	-0.21±0.04
[6]DD_{asym}	p=0.1	q=0.6	6.1±0.6	-1.8±0.05	0.035±0.004	0.64±0.05	0.24±0.032	-0.14±0.06	-0.69±0.04
[7]$DD_{asym-rw}$	p=0.1	q=0.6	8.5±0.5	-1.8±0.05	0.035±0.002	0.44±0.02	0.20±0.026	0.08±0.02	-0.56±0.03
AP	-		8.0±0.0	-3.3±0.2	0.007±0.0004	0.34±0.004	0.018±0.001	0.37±0.02	0.47±0.02
CG	Newman		8.0±0.0	-2.83±0.06	0.28±0.007	0.77±0.02	1.01±0.03	-0.13±0.02	0.50±0.10
	TCC		8.0±0.0	-2.82±0.06	0.27±0.006	0.77±0.02	1.01±0.02	-0.14±0.02	0.51±0.16

Each model is run 100 times until the number of nodes reaches 3000. The average degree is constant (<k>=8.0) for PA, AP and CG model but is variable in DD model depending on the parameters p and q.

[1] In DD model, p=the connection probability between duplicates, q=the divergence probability. In CG model, the module is defined by the method by Newman or TCC (triangularly connected component).

[2] average degree

[3] the degree exponent in $P(k) \sim k^{-\gamma}$

[4] the number of triangles per edge

[5] the mixing exponent in $\langle k_{nn} \rangle (k) \sim k^{-\delta}$

[6] Ispolatov et al. (2005) Phys Rev E Stat Nonlin Soft Matter Phys 71: 061911

[7] Pastor-Satorras et al. (2003) J Theor Biol 222: 199-210. (simulated using the equation 17)
